# A systematic review of online education initiatives to develop students remote caring skills and practices

**DOI:** 10.1080/10872981.2022.2088049

**Published:** 2022-06-12

**Authors:** Lorelli Nowell, Swati Dhingra, Sandra Carless-Kane, Claire McGuinness, Alessandra Paolucci, Michele Jacobsen, Diane L. Lorenzetti, Liza Lorenzetti, Elizabeth Oddone Paolucci

**Affiliations:** a Faculty of Nursing, University of Calgary, Calgary, AB, Canada; b Werklund School of Education, University of Calgary, AB, Canada; c Department of Community Health Sciences, Cumming School of Medicine, University of Calgary, AB, Canada; d Health Sciences Library, University of Calgary, AB, Canada; e Faculty of Social Work, University of Calgary, Calgary, AB, Canada; f Department of Surgery, Cumming School of Medicine, University of Calgary, AB, Canada

**Keywords:** Caring professions, innovation, online education, remote care, systematic reviews

## Abstract

The ongoing COVID-19 pandemic has altered caring professions education and the range of technological competencies needed to thrive in today’s digital economy. We aimed to identify the various technologies and design strategies being used to help students develop and translate professional caring competencies into remote working environments. Eight databases were systematically searched in February 2021 for relevant studies. Studies reporting on online learning strategies designed to prepare students to operate in emerging digital economies were included. Quality assessment was undertaken using the Effective Public Health Practice Project Quality Assessment Tool and/or the Joanna Briggs Institute Critical Appraisal Checklist for Qualitative Research. Thirty-eight studies were included and synthesized to report on course details, including technologies being used and design strategies, and study outcomes including curriculum, barriers and facilitators to technology integration, impact on students, and impact on professional practice. Demonstrations of remote care, videoconferencing, online modules, and remote consultation with patients were the most common instructional methods. Audio/video conferencing and online learning systems were the most prevalent technologies used to support student learning. Students reported increased comfort and confidence when working with technology and planning and providing remote care to patients. While a recent influx in research related to online learning and caring technologies was noted, study quality remains variable. More emphasis on assessment, training, and research is required to support students in using digital technologies and developing interpersonal and technological skills required to work in remote settings.

Caring professions are occupations where individuals prioritize the health, educational, and social needs of society rather than its material needs; examples of such include Education, Medicine, Nursing, Social Work, and other Allied Health professions [1]. The various roles and functions of caring professions have been crucial catalysts in shaping and influencing the global response to the COVID-19 pandemic. However, in unprecedented and unpredictable ways, this global crisis has also significantly altered the ways in which education is delivered and care is provided, requiring caring professions and higher education institutions to ensure that core caring competencies vital to care provision, continue to be taught and implemented in increasingly virtual environments [2–5]. Education and caring profession practices are often provided face-to-face, where body language, emotional cues, and compassion may be more easily and naturally communicated and experienced. Specialized technologies are increasingly being created and integrated into practice settings to enable caring professions to provide care effectively in virtual settings. It is therefore necessary to design coherent, evidence-based strategies that support students in these professions to adapt to the evolving technological requirements of their roles and develop and translate professional caring competencies to digital working environments [6].

The aim of this study was to conduct a systematic review of the prevalence and diversity of technologies and design strategies used to prepare graduates in caring professions for competent and effective practice in digital working environments. Our multidisciplinary review will help identify strengths and areas for future growth within caring professions education. This review provides direction from across caring disciplines; specifically, those that instil social, technical, and communication skills development to manage and maintain interpersonal relationships as central to their profession. Caring professions are hands-on, practice-focused, public-facing professions and this review provides an opportunity to explore the transferability of the knowledge and strategies within these practice-based caring professions. The research question that guided this review was: *What technologies and design strategies are being used to prepare caring professions students to operate in virtual caring environments?*

## Methods

We conducted a systematic review, including qualitative, quantitative, and mixed methods studies, in accordance with Joanna Briggs Institute and PRISMA guidelines for the conduct and reporting of systematic reviews [7,8]. The study protocol was peer-reviewed and published by BMJ Open on 19 May 2021 to ensure transparency in our process [9].

### Search Strategy

A comprehensive search of eight databases was conducted in February 2021 in compliance with the Preferred Reporting Items for Systematic Reviews and Meta-Analyses (PRISMA) guidelines [8]. We searched CINAHL, Education Research Complete, EMBASE, ERIC, MEDLINE, Scopus, Social Work Abstracts, and Web of Science databases for eligible studies. As well, snowball searching was completed, where the references and cited-by’s of included studies were screened for additional studies. Full search strategies for each database are available in Supplementary Table 1.

### Eligibility criteria

Study eligibility criteria are highlighted in Table 1. We included qualitative, quantitative and mixed-methods studies that met our eligibility criteria. We excluded studies published over 10 years ago to capture only the most recent and relevant online technologies, pedagogies, and practices.

**Table 1.** Eligibility criteria

Inclusion criteria	Exclusion criteria
Focused on the education of undergraduate and/or graduate students in the caring profession disciplines Described current strategies to offer online learning designed to prepare students to operate in emerging digital economies Reported on the impact of implementing these strategies including student and teacher perspectives, learning outcomes, capacity of students to develop career skills and competencies, and patient or learner perspectives	Focused on continuing education of professionals currently in practice Commentaries, editorials, letters or non-systematic reviews that do not report on outcomes or impact associated with online education Published over 10 years ago Non-English language studies

### Study selection and data extraction

**Figure 1.**
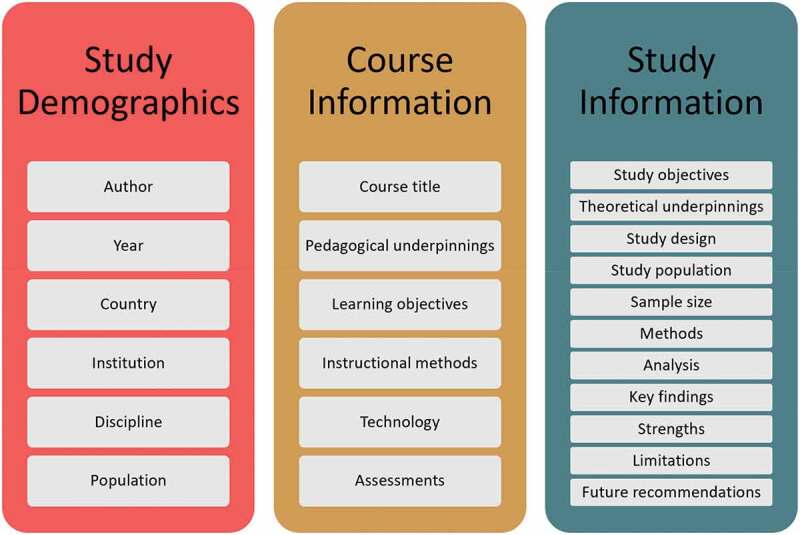
Data extraction items.

Teams of two reviewers independently screened study titles and abstracts, and full text, in duplicate. Areas of discordance were resolved through discussion or by a third reviewer. We maintained an overall interrater agreement above 90% throughout the screening process. A standardized Excel data extraction tool was used, where one reviewer extracted study data and a second reviewer verified the extracted data for accuracy. The data items extracted are displayed in Figure 1.

### Quality assessment

To maintain consistency in our approach, teams of two reviewers independently assessed the quality of all included studies in duplicate and any areas of discordance were resolved through discussion or by a third reviewer. We used the Effective Public Health Practice Project Quality Assessment Tool (EPHPP) to rate each quantitative study as strong, moderate, weak, or not applicable across six domains of validity and reliability [10]. We used the Joanna Briggs Institute Critical Appraisal Checklist for Qualitative Research [7] to rate each qualitative study across ten domains requiring a yes, no, unclear, or not applicable response [11]. For mixed methods studies, we used both the appraisal tools.

### Data synthesis

The identified studies varied considerably in aim(s), technologies, design strategies, study design, course learning objectives, and outcomes. We utilized a convergent qualitative synthesis design [12] to transform quantitative and mixed methods studies into qualitative findings and integrated them with the qualitative narrative synthesis using a Bayesian approach [13]. In this process, an overarching synthesis was created where both quantitative and qualitative data were given equal weight to capture all outcomes identified in the studies. Data were thematically analysed [14,15] by a process of induction to transform data from individual studies to common, interactive themes [16]. While we proposed to conduct a sensitivity analysis to examine the influence of studies with a low-quality rating on the robustness of review findings, the overall low quality of studies identified prevented this comparison.

## Results

**Figure 2.**
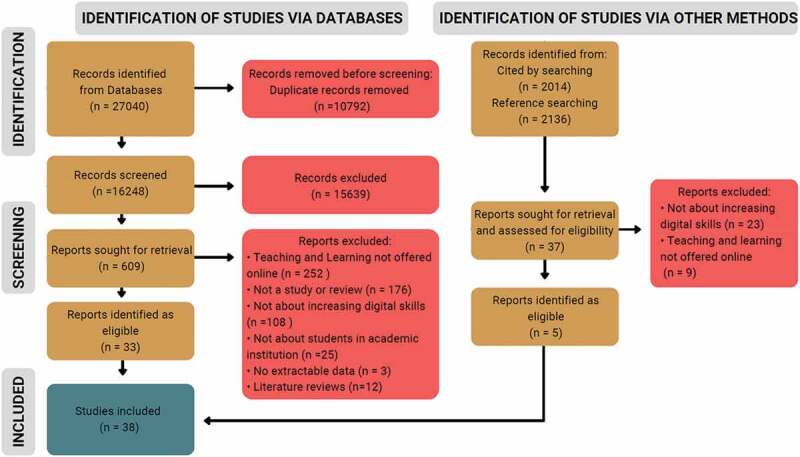
PRISMA flow diagram.

A total of 16,248 unique studies were identified through database and other manual searching strategies, which were then subsequently screened for inclusion, 609 of which underwent secondary full-text screening. Of these, 38 studies were included in the final synthesis. Figure 2 displays the flow of literature throughout our review.

### Study characteristics

**Figure 3.**
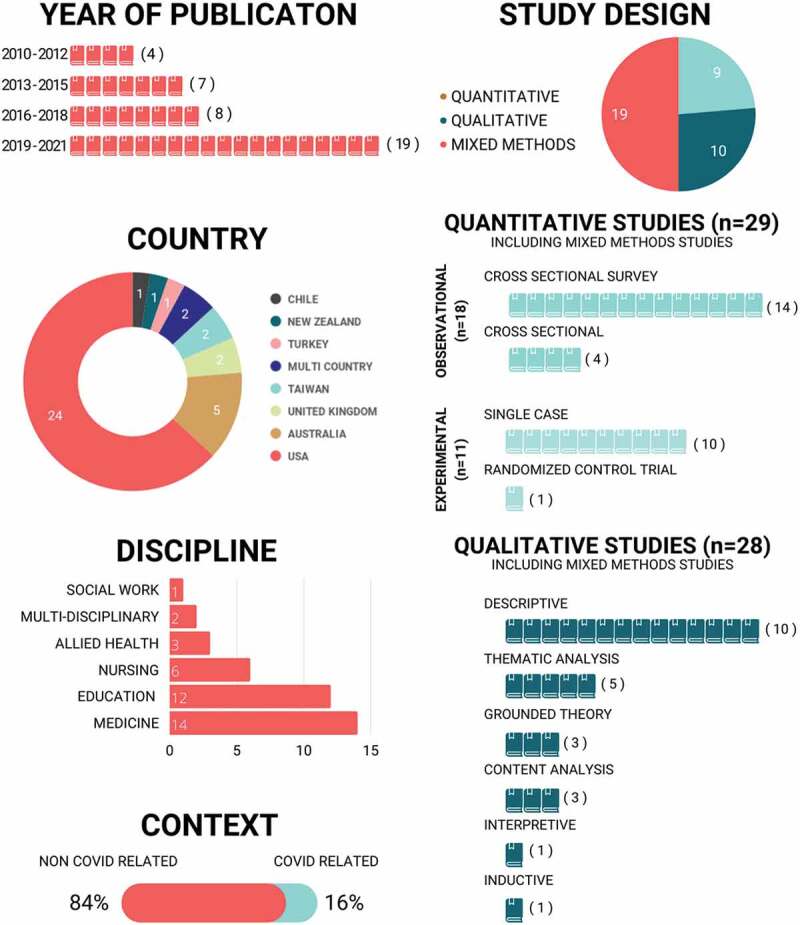
Study characteristics.

Figure 3 provides a high-level overview of the study characteristics. Fifty percent (n = 19) of studies were published in the past three years and 63% (n = 24) originated in the US. Most studies in this synthesis were focused on medicine (n = 14, 37%), education (n = 12, 32%) and nursing (n = 6, 16%). Fifty percent (n = 19) of the studies utilized a mixed-methods study design. Including the mixed-methods studies, 29 (76%) studies utilized quantitative methods and 28 (74%) studies utilized qualitative methods. Detailed study information is provided in Supplementary Table 2.

### Quality appraisal

The quality appraisal scores for all studies are presented in supplementary Tables 3 and 4. Overall, quantitative studies were appraised to be weak due to their design (n = 24, 83%), absence of blinding (n = 29, 100%), and data collection methods (n = 21, 72%). Qualitative studies were appraised to be weak due to incongruent methods (n = 6, 21%), unclear interpretations of results (n = 13, 46%), and incongruent conclusions (n = 8, 28%).

### Courses

**Figure 4.**
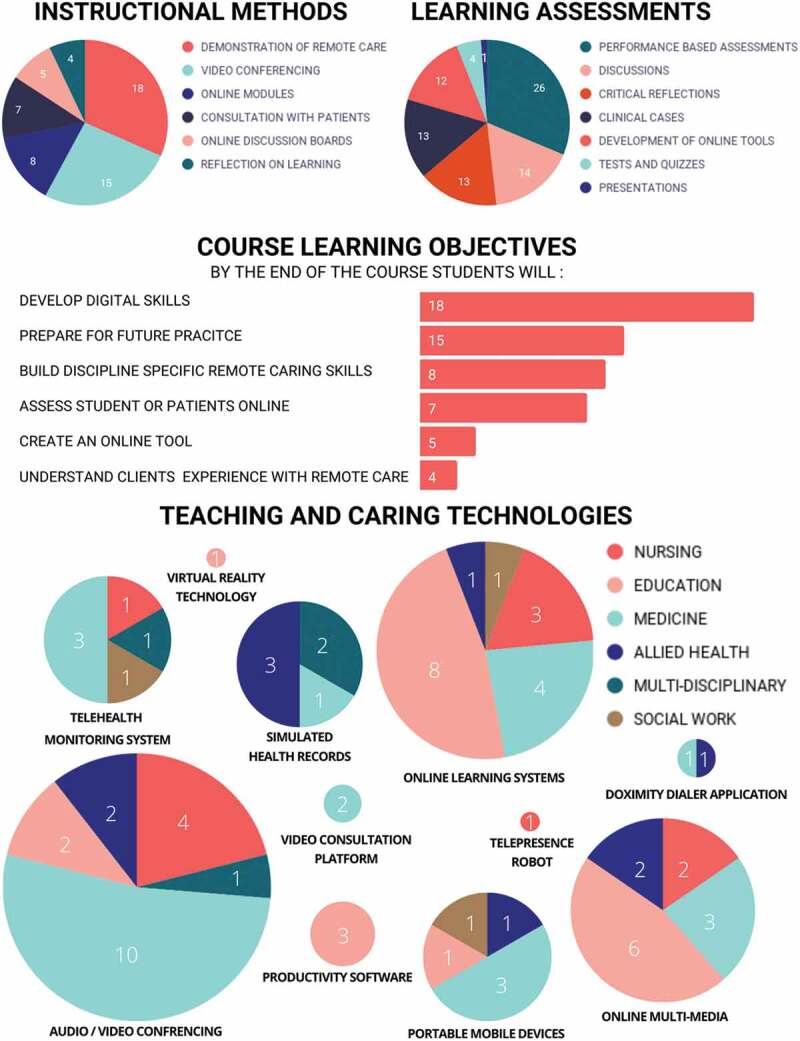
Summary of course objectives, instruction, assessment and technologies.

Figure 4 provides a high-level overview of the courses studied including the instructional methods, learning assessments, course learning objectives, and technologies used. The length of the courses ranged from 2 hours to 18 weeks.

#### Instructional methods

More than half (n = 21, 55%) of the studies used more than one instructional method and nine (24%) studies implemented three or more instructional methods. Demonstrations of remote care was the most widely used instructional method (n = 18, 47%). Of the 18 studies that utilized demonstrations of remote care, most of them were in the disciplines of Medicine or Nursing except for O’Connor and Worman (2019) in the discipline of Education. Videoconferencing was the second most highly used instructional method (n = 15, 39%) in the disciplines of Medicine, Nursing, and Education. Eight studies [21%) incorporated online modules, with 17,solely using online modules as an instructional method. Seven studies [38%] utilized consultation with patients, with 18,solely using consultation as an instructional method. Five studies (13%] utilized online discussion boards in conjunction with other modes of instruction. These five studies were in the discipline of Medicine, Education and Social Work. Similarly, four studies (10%) from Medicine and Social Work incorporated reflections on learning in conjunction with other modes of instruction.

#### Learning assessments

Seven types of learning assessments emerged from the extracted data. Clinical Case Assessment strategies included: chapter reviews, consults, image-based diagnosis, interviews (e.g., patient-structured), reasoning and diagnosis exercises, and tutorials (e.g., diagnosis and management). Critical Reflection Assessments strategies required students to complete autobiography, biography, blog, journal, paper, and/or discussion posts. Additionally, critical reflections also required students to reflect on a video, complete a survey, and/or in engage in self and/or peer debriefing. Studies also required students to develop online course content including creating online courses, lesson plans, multimedia, networks (e.g., Personal Learning Network), questions and answer index/web cards, social media resources, stories, and teaching resources either individually or in an online group setting. Discussion assessments were active and online, and usually involved e-consults, students providing feedback, group discussions, and/or learning community forums. Students were most commonly assessed using performance-based measures in which they demonstrated how they could use the technology in real-world or simulated settings (n = 26, 68%). Performance-based assessments involved: assessing and/or interviewing simulated or standardized patients; design thinking projects; documentation of patient concerns; engaging in virtual encounters and simulations; multiple-choice and open-ended tests or quizzes; patient consultations and/or tele-visits; patient follow-up notes; patient history and physical exam; practicum; OSCEs; research; teaching (e.g., direct, observation table, philosophy synthesis); triage; and using technical equipment. Only one (3%) study [19] incorporated presentations as a learner assessment strategy.

#### Course learning objectives

The most common learning outcomes were: development of digital skills (n = 18, 47%) and preparation for future online practice (n = 15, 39%). Less than a quarter (n = 18, 21%) of the courses focused on building students’ discipline-specific remote care skills. While some courses sought to give student opportunities to assess students and/or patients online (n = 7, 18%), only four studies (10%) aimed to teach students about patients’ experiences with remote care.

#### Teaching and caring technologies

All included studies incorporated a form of online technology. Fourteen (37%) studies implemented one type of technology, and twenty-three [61%) studies used more than one technology. 20,in the discipline of Education most extensively utilized seven various forms of technology. Audio/Video Conferencing was the most widely used teaching and learning technology (n = 19, 50%] which often included Google Meet, Microsoft Teams, and Zoom. Moreover, Online Learning Systems was the second most highly implemented teaching and learning technology (n = 17, 44%) and typically comprised the following: the Moodle Learning Platform, Synchronous Learning Management System, the Blackboard Learning Management System, Online Forum and Discussion Boards, Online Modules, Web Based Software Tools (e.g., CASUS Authoring System, WebQuest, Wiki), and Web Based Tele-Education System (e.g., Virtual OSCE cases). Aside from these two most used technologies, three overarching categories also surfaced from this review including Online Multimedia, Productivity Software, and Portable/Mobile Devices. Online Multimedia involved audio, video, and/or screen recordings (e.g: Audacity, Camera Software, Digital Microscopes, GarageBand, Images, Interactive Whiteboard Animation Software, Wimba Voice, and YouTube Clips). Additionally, Productivity Software included the use of Email, Information and Communication Technology, Microsoft Word and PowerPoint. Despite online environments requiring the use of a portable or mobile device, few (n = 6, 16%) studies mentioned the use of Portable/Mobile Devices (e.g., telephones, smartphones, tablets, and laptops), and only two (5%) studies revealed using Video Consultation Tools (e.g: AccuRx, Internet Dataloggers, Simulated Academic Electronic Health Records (EHR), Video Editing Software, and Virtual Reality Technology); these were the least incorporated or referenced forms of technology.

### Outcomes

#### Curriculum

The onset of COVID-19 required curriculum adaptations including the development of new remote and online consultations, physical exams, and telehealth learning opportunities [19, 21–27. Even prior to COVID-19 researchers reported that many students appreciated the incorporation of innovative and creative pedagogical strategies to support their learning about remote technology (28]. When learning activities were in alignment and closely replicated real-world situations, authors noted that students viewed these as more helpful and effective at preparing them for future practice [19,29,30]. The design of online learning activities affected the level of student motivation and participation [31]. Researchers across studies reported that learning activities need to be thoughtfully designed specifically in terms of organization, workload, and time for both instructors and students [31,32].

#### Technology Integration

From a teacher’s perspective, utilizing digital technology in classrooms was found to increase cost, time, energy, and effort, all of which may impede online teaching and learning experiences [33]. Further, researchers reported that learning new technologies for teaching could be tedious and frustrating [20], and technical problems and limitations could interfere with interactions that would normally occur face-to-face [29, 34–36; 37]. While teachers’ level of confidence with integrating online approaches into the classroom was noted as a potential barrier for technology integration [38], 39,suggested that despite the practical and educational challenges that many new technologies bring, when faculty take an intentional approach towards addressing these challenges, such challenges can be overcome.

There were also barriers to technology integration and use noted from the students’ perspectives. Cantone and colleagues reported that students may worry about how they will be assessed when trying something new [34]. Designs that lacked opportunities for human interaction was also noted as a key factor that could negatively affect students’ professional, social, and personal development [27]. Researchers found that when the technology used in online classrooms was not viewed as relevant, students became skeptical of its utility and viability [34] and struggled to envision its use in professional practice [20]. Finally, researchers also reported that remote teaching and caring can limit the scope and reliability of assessment capabilities which can foster a distanced and impersonal experience for students [29,35].

#### Impact on Student Learning

The most noted impact on students was an increase in confidence in using digital technology [23,32,38,40]. Students reported increased comfort and confidence when working with telehealth technology and planning and providing telehealth to patients [25, 26, 33, 41, 42; 19, 30, 43, 44]. Student teachers also demonstrated increased comfort creating online learning environments [17,18,37,45].

Researchers reported that several elements of virtual communications differed from in-person engagement including lighting, body positioning and movements, and the use of silence to communicate empathy and concern [41]. Students gained an appreciation for the differences between in-person and remote care and teaching [25,34] and recognized the need to increase connectivity and communication despite the distance factor [46]. Students also learned strategies for interacting in online environments in a respectful manner and practicing with integrity as a key concept [47; 19], with several studies noting improvements in students’ communication skills [21,29,32,48]. Only one study reported a decrease in confidence in using appropriate professional language when interacting with patients in an online environment [30].

Students generally valued opportunities to apply and practice clinical skills with real-world technology outside of a high-stakes testing environment [25,49] and benefited from direct observation of skills and immediate faculty and peer feedback [33,41]. That said, researchers did note that some students found it challenging to learn remote skills in an online or simulated environment [31,36,44,49].

#### Impact on Professional Practice

Several studies highlighted the potential impacts of students learning and using technology to care and teach remotely on future caring professions practice. In general, students appreciated learning how to conduct remote care as a key competence for their future practice [22,32,39,47,49]. As a result of engaging in remote care teaching and learning, many students suggested that they were likely to teach and/or develop an online learning opportunity in the future [18] or incorporate virtual visits into their future practice [26].

## Discussion

This systematic review was undertaken to identify the various technologies and design strategies used to help students develop and translate professional caring competencies into remote working environments. This study provides a unique multi-disciplinary contribution to the existing literature. A detailed review of 38 eligible studies identified a myriad of learning technologies being employed as pedagogical strategies within the caring professions, from online videos and demonstrations to telehealth monitoring and virtual platforms for clinical assessments. Our study revealed that purposeful integration of online digital technologies can have a positive impact on students and their development of caring professions practices. Overall, the findings from the studies indicate that student participants embraced the benefits of these approaches and expressed and/or demonstrated increased comfort and competency when translating this knowledge into practice.

Our findings reveal that in recent years (i.e., 2019–2021) there has been an influx of research conducted in caring disciplines related to integrating online learning opportunities to build remote caring skills and practices. While research is being conducted worldwide, our synthesis shows the amalgam of emerging studies on caring professions’ competencies are being undertaken primarily in the USA, with growing collaborations across multiple countries. Medicine has produced the most research in this area compared to other disciplines, with an increasing number of multidisciplinary studies being published in recent years. This may indicate that integrating effective caring practices for students across caring disciplines is a future focused strategy for cohesive translation of remote caring into our digital economy.

The quality of identified studies tended to be weak or incongruent with the research methodology adopted, suggesting room for improvement in the areas of appropriate sampling, blinding researchers to group identity, controlling for or addressing confounding variables, and reporting attrition rates (i.e., quantitative studies), and better inclusion of philosophy, reflexivity statements, and ethical considerations (i.e., qualitative studies). Alternatively, the lower quality score ratings may also be due to the original assumptions guiding the development of the quality appraisal tools that were used in our review, and their application to disciplines that are less likely to employ higher level evidence study design types. For instance, in quantitative studies, random sampling procedures are considered to produce the strongest level of evidence, but there are several reasons why researchers may be unable to achieve this standard when conducting research in the social sciences and education disciplines (i.e., ethical and relevance constraints related to employing experimental study designs, ontological and epistemological assumptions about learning, recruitment challenges, smaller resources available from funding agencies, and so on).

The learning objectives from the studies identified in our review suggest that caring professions are working towards building knowledge of digital skills and preparing students for future practice that incorporates the innovations of online and simulated technology. This finding aligns with the substantial advancements that have been made in recent years to prioritize the development of novel technologies for future health professionals [50]. A variety of technologies used in caring professions were identified in our review. Several technologies were used to support student learning while also providing a means of assessment, demonstrating how assessment techniques often require the use of technology and how our digital economy has merged the two. When teaching students online, instructors often included videoconferencing, demonstrations, role-plays, and encounters with standardized patients to combine online lecture-based learning with practical and skill-based components [51]. Moreover, this intersection between education and technology enables students to readily access a wealth of information and resources in today’s digital environment [52]. While researchers have suggested that online learning requires a balance between theory and practice tasks [51], this may not yet have been fully realized when supporting students to use technology for remote care and teaching.

As a result of the COVID-19 pandemic and as evident from our review, teaching, learning, and assessment has proven possible in the caring professions with the exclusive use of technology. In particular, education for caring competencies in the caring disciplines has relied largely on audio and videoconferencing software (e.g., Zoom) and other online learning systems (e.g., Desire 2 Learn) to facilitate remote course learning and assessments for students. While these technologies enable teacher and student communication and connection, they may not always allow students to participate in all areas of their knowledge and skill development and this calls for various features inherent within online technology (e.g., telehealth system) to support student learning.

Exploration of newer methods to successfully integrate telehealth into educational and clinical settings have been frequently identified across the nursing and medicine literature suggesting this is an ongoing priority [53–57]. Considering the recognized importance of telehealth education across health professions, its integration into the academic curriculum would enhance the skills of healthcare students to successfully function in today’s digital world. Identifying more unified digital platforms and ways of facilitating student learning, across all caring disciplines, can foster robust knowledge and skill development that may aid them in applying caring practices in the current and evolving digital economy.

### Implications for practice

Through our review we identified opportunities for further advancements in online learning to support digital and caring competencies across caring professions. Our findings suggest that more emphasis, assessment, and training is required for faculty to immerse students in using digital online collaboration tools and developing interpersonal and technological skills that prepare them for work in remote and virtual settings. Specifically, better mechanisms are needed to ensure students use technology across all subject areas during their placements, consistent with current technology standards. Second, creating opportunities for professional practice leaders across organizations and educators in higher educational settings to collaborate and integrate high impact online learning experiences may better prepare graduates for practice. Third, promoting assessment and feedback efforts at the institutional level could further extend understanding of online instruction and provide learners with more opportunities to contribute to the improvement of teaching and learning experiences. Fourth, there is a need to continue generating suitable strategies for online placements as opportunities to advance learning and skills-building in remote and virtual environments. Finally, a framework and/or benchmarks are needed to understand the conditions that may enhance productive e-learning opportunities.

### Suggestions for future research

Our findings highlight some key areas for future research. First, more rigorous examination of the experiences of students and faculty with technology and online courses is warranted. Future qualitative studies should explore perceptions and experiences of students in different online environments to further our understanding of participation in such settings, including motivation, group dynamics and skills development. Second, evaluation of the design and delivery aspects of online courses intended to increase digital competence of students in caring professions, and the factors affecting technology integration in online courses are important areas for further exploration. Specifically, research is needed to explore the interrelationship between task design and participation in online settings. Similarly, there is a need to identify and explore the distinctive aspects of the virtual experience, particularly during pandemics. We suggest a robust and comprehensive evaluation of these factors related to technology integration and design of online courses for future studies. Finally, more longitudinal research is needed to examine the persistence of students’ practices of incorporating technology in their professional practice, as well as more evidence assessing the practical results of training on student performance in real world-settings.

## Limitations

While we conducted this review using rigorous and established methods, several limitations remain inherent. First, while we aimed to robustly and comprehensively identify the various technologies and design strategies being used to help students develop and translate professional caring competencies into remote working environments, a search of the grey literature may have identified additional evidence of relevance to this review. Second, most of the included studies were from the USA; while this reflects the current state of evidence, this disproportionate geographical representation may not accurately reflect standard practices in higher education institutions worldwide. Third, the methodological quality of the included studies was rated weak to moderate, so we were unable to make inferences about which strategies are most successful in preparing students to operate effectively in digital economies.

Despite these limitations, the findings from this review reflect the current state of evidence for innovative online education initiatives used to prepare graduates in caring professions for employment, and competent and effective practice in the digital economy. Our findings also underscore the need for additional research, with robust study designs, to better understand the impact and outcomes of innovative online education initiatives for graduates in caring professions.

## Conclusion

This review identified the ways in which educators in caring professions can design and integrate online learning opportunities to help students develop and translate caring competencies into digital working environments. The results of this knowledge synthesis can inform the more effective design of online learning opportunities to support caring professions’ digital competency development amid ongoing change and complex contexts.

## Supplementary Material

Supplemental Material
