# Supplementary material for: A systematic review of online education initiatives to develop students remote caring skills and practices
Source: Med Educ Online. 2022 Jun 12;27(1):2088049. doi: 10.1080/10872981.2022.2088049 (PMC9196772; doi:10.1080/10872981.2022.2088049)
Supplement: Supplemental Material [file ZMEO_A_2088049_SM7708.docx]

**Supplementary Table 1**

*Search Strategy*

| **Database** | **Search Strategy** | **Limiters** | **Total** |
| --- | --- | --- | --- |
|  |  |  |  |
| CINAHL | 1. (MH "Education, Allied Health+") OR (MH "Education, Nursing, Baccalaureate+") OR (MH "Education, Nursing, Graduate+") OR (MH "Education, Nurse Midwifery") OR (MH "Education, Nursing") OR (MH "Education, Medical") OR (MH "Education, Chiropractic") OR (MH "Education, Pharmacy") OR (MH "Education, Podiatry") OR (MH "Education, Nursing, Diploma Programs")  2. TI ( ((allied health or clinician* or doctor* or health profession* or medical student* or nurs* or paremedic* or physician* or psycholog* or psychiatr* or social work* or teacher*) N3 (educat* or train*)) ) OR AB ( ((allied health or clinician* or doctor* or health profession* or medical student* or nurs* or paremedic* or physician* or psycholog* or psychiatr* or social work* or teacher*) N3 (educat* or train*)) )  3. 1or 2  4. (MH "Telehealth+") OR (MH "Educational Technology") OR (MH "Informatics") OR (MH "Medical Informatics") OR (MH "Nursing Informatics") OR (MH "Therapy, Computer Assisted+")  5. TI ( ((care or consultation* or educat* or healthcare or instruction or learning or teaching) N3 (computer* or digital or electronic or online)) ) OR AB ( ((care or consultation* or educat* or healthcare or instruction or learning or teaching) N3 (computer* or digital or electronic or online)) )  6. TI ( (digital therapeutic* or digital technolog* or ehealth or e-health or e-support* or information technolog* or mobile health or mhealth or m-health or remote consult* or teleconsult* or tele-consult* or telehealth or tele-health* or telemedic* or tele-medic* or telepsychiatr* or tele-psychiatr* or teletherap* or tele-therap*) ) OR AB ( (digital therapeutic* or digital technolog* or ehealth or e-health or e-support* or information technolog* or mobile health or mhealth or m-health or remote consult* or teleconsult* or tele-consult* or telehealth or tele-health* or telemedic* or tele-medic* or telepsychiatr* or tele-psychiatr* or teletherap* or tele-therap*) )  7. TI digital econom* OR AB digital econom*  8. TI ( ((education* or information*) N3 technolog*) ) OR AB ( ((education* or information*) N3 technolog*) )  9. 4 or 5 or 6 or 7 or 8  10. 3 and 9 | Published Date: 20100101-20211231; Scholarly (Peer Reviewed) Journals; English Language | 4681 |
| Education Research Complete | 1. ((DE "TEACHER education") OR (DE "MEDICAL education" OR DE "ALLIED health education" OR DE "NURSING education" OR DE "PSYCHIATRY education")) OR (DE "SOCIAL work education")  2. TI ( ( ((allied health or clinician* or doctor* or health profession* or medical student* or nurs* or paremedic* or physician* or psycholog* or psychiatr* or social work* or teacher*) N3 (educat* or train*)) ) ) OR AB ( ( ((allied health or clinician* or doctor* or health profession* or medical student* or nurs* or paremedic* or physician* or psycholog* or psychiatr* or social work* or teacher*) N3 (educat* or train*)) ) )  3. 1 or 2  4. (DE "EDUCATIONAL technology" OR DE "PROGRAMMED instruction" OR DE "COMPUTERS in education" OR DE "INSTRUCTIONAL systems" OR DE "VIRTUAL classrooms") OR (DE "COMPUTER assisted instruction" OR DE "ONLINE education" OR DE "BLENDED learning" OR DE "COMPUTER managed instruction")  5. TI ( ( ((care or consultation* or educat* or healthcare or instruction or learning or teaching) N3 (computer* or digital or electronic or online)) ) ) OR AB ( ( ((care or consultation* or educat* or healthcare or instruction or learning or teaching) N3 (computer* or digital or electronic or online)) ) )  6. TI ( ( (digital therapeutic* or digital technolog* or ehealth or e-health or e-support* or information technolog* or mobile health or mhealth or m-health or remote consult* or teleconsult* or tele-consult* or telehealth or tele-health* or telemedic* or tele-medic* or telepsychiatr* or tele-psychiatr* or teletherap* or tele-therap*) ) ) OR AB ( ( (digital therapeutic* or digital technolog* or ehealth or e-health or e-support* or information technolog* or mobile health or mhealth or m-health or remote consult* or teleconsult* or tele-consult* or telehealth or tele-health* or telemedic* or tele-medic* or telepsychiatr* or tele-psychiatr* or teletherap* or tele-therap*) ) )  7. TI digital econom* OR AB digital econom*  8. TI ( ( ((education* or information*) N3 technolog*) ) ) OR AB ( ( ((education* or information*) N3 technolog*) ) )  9. 4 or 5 or 6 or 7 or 8  10. 3 and 9 | Scholarly (Peer Reviewed) Journals; Published Date: 20100101-20211231; Language: English | 5557 |
| Embase  *1974-2021* | 1. medical education/ or physician assistant education/ or residency education/ (245007)  2. nursing education/ or nurse anesthesia education/ or nurse midwifery education/ (83492)  3. teacher training/ (538)  4. social work education/ (100)  5. paramedical education/ or allied health education/ or chiropractic education/ or dietetics education/ or emergency medical services education/ or midwifery education/ or nurse training/ or occupational therapy education/ or physical therapy education/ or podiatry education/ (7732)  6. ((allied health or clinician* or doctor* or health profession* or medical student* or nurs* or paramedic* or physician* or psycholog* or psychiatr* or social work* or teacher*) adj3 (educat* or professional development or train*)).tw,kw. (129168)  7. 1 or 2 or 3 or 4 or 5 or 6 (416119)  8. exp telemedicine/ (46166)  9. educational technology/ (3147)  10. computer assisted therapy/ (4751)  11. medical informatics/ or nursing informatics/ (22518)  12. ((care or consultation* or educat* or healthcare or instruction or learning or teaching) adj3 (computer* or digital or electronic or online)).tw,kw. (24531)  13. (digital therapeutic* or digital technolog* or ehealth or e-health or e-support* or information technolog* or mobile health or mhealth or m-health or remote consult* or teleconsult* or tele-consult* or telehealth or tele-health* or telemedic* or tele-medic* or telepsychiatr* or tele-psychiatr* or teletherap* or tele-therap*).tw,kw. (62978)  14. digital econom*.tw,kw. (52)  15. ((education* or information*) adj3 technolog*).tw,kw. (28247)  16. 8 or 9 or 10 or 11 or 12 or 13 or 14 or 15 (140044)  17. 7 and 16 (13713)  18. limit 17 to english language (12976)  19. limit 18 to yr="2010 -Current" (8208)  20. limit 19 to conference abstracts (1859)  21. 19 not 20 (6349)  22. limit 21 to (editorial or letter) (509)  23. 21 not 22 (5840)  24. limit 23 to "systematic review" (161)  25. ((critical or evidence-based or integrative or rapid or realist or scoping or systematic or umbrella) adj (overview* or review* or synthes*)).tw,kw. (278612)  26. (metanalys* or metaanalys* or meta-analys*).tw,kw. (252127)  27. 25 or 26 (419500)  28. 23 and 27 (271)  29. 24 or 28 (311)  30. limit 23 to "review" (763)  31. 23 not 30 (5077)  32. 29 or 31 (5248) |  | 5248 |
| ERIC | 1. (DE "Teacher Education") AND (DE "Medical Education" OR DE "Graduate Medical Education" OR DE "Nursing Education" OR DE "Pharmaceutical Education" OR DE "Allied Health Occupations Education")  2. TI ( ( ((allied health or clinician* or doctor* or health profession* or medical student* or nurs* or paremedic* or physician* or psycholog* or psychiatr* or social work* or teacher*) N3 (educat* or train*)) ) ) OR AB ( ( ((allied health or clinician* or doctor* or health profession* or medical student* or nurs* or paremedic* or physician* or psycholog* or psychiatr* or social work* or teacher*) N3 (educat* or train*)) ) )  3. 1 or 2  4. (((((DE "Educational Technology" OR DE "Online Courses") OR (DE "Computer Assisted Instruction")) OR (DE "Virtual Classrooms")) OR (DE "Computer Mediated Communication")) OR (DE "Blended Learning")) AND (DE "Computer Uses in Education" OR DE "Technology Integration" OR DE "Technology Uses in Education" OR DE "Web Based Instruction")  5. TI ( ( ((care or consultation* or educat* or healthcare or instruction or learning or teaching) N3 (computer* or digital or electronic or online)) ) ) OR AB ( ( ((care or consultation* or educat* or healthcare or instruction or learning or teaching) N3 (computer* or digital or electronic or online)) ) )  6. TI ( ( (digital therapeutic* or digital technolog* or ehealth or e-health or e-support* or information technolog* or mobile health or mhealth or m-health or remote consult* or teleconsult* or tele-consult* or telehealth or tele-health* or telemedic* or tele-medic* or telepsychiatr* or tele-psychiatr* or teletherap* or tele-therap*) ) ) OR AB ( ( (digital therapeutic* or digital technolog* or ehealth or e-health or e-support* or information technolog* or mobile health or mhealth or m-health or remote consult* or teleconsult* or tele-consult* or telehealth or tele-health* or telemedic* or tele-medic* or telepsychiatr* or tele-psychiatr* or teletherap* or tele-therap*) ) )  7. TI digital econom* OR AB digital econom*  8. TI ( ( ((education* or information*) N3 technolog*) ) ) OR AB ( ( ((education* or information*) N3 technolog*) ) )  9. 4 or 5 or 6 or 7 or 8  10. 3 and 9 | Peer Reviewed; Date Published: 20100101-20211231; Language: English | 3735 |
| Ovid MEDLINE  1946-2021 | 1. exp education, medical, graduate/ or education, medical, undergraduate/ or education, nursing/ or education, nursing, baccalaureate/ or education, nursing, diploma programs/ or education, nursing, graduate/ or teacher training/ (153349)  2. exp Allied Health Personnel/ed [Education] (14103)  3. Nurse Midwives/ed [Education] (1409)  4. exp Nurse Practitioners/ed [Education] (3283)  5. exp Social Workers/ed [Education] (83)  6. ((allied health or clinician* or doctor* or health profession* or medical student* or nurs* or paremedic* or physician* or psycholog* or psychiatr* or social work* or teacher*) adj3 (educat* or train*)).tw,kf. (102364)  7. 1 or 2 or 3 or 4 or 5 or 6 (242065)  8. Telemedicine/ or Educational Technology/ or informatics.tw,kf. (42828)  9. remote consultation/ (5016)  10. exp Therapy, Computer-Assisted/ (66790)  11. ((care or consultation* or educat* or healthcare or instruction or learning or teaching) adj3 (computer* or digital or electronic or online)).tw,kf. (17453)  12. (digital therapeutic* or digital technolog* or ehealth or e-health or e-support* or information technolog* or mobile health or mhealth or m-health or remote consult* or teleconsult* or tele-consult* or telehealth or tele-health* or telemedic* or tele-medic* or telepsychiatr* or tele-psychiatr* or teletherap* or tele-therap*).tw,kf. (52359)  13. digital econom*.tw,kf. (39)  14. ((education* or information*) adj3 technolog*).tw,kf. (23217)  15. 8 or 9 or 10 or 11 or 12 or 13 or 14 (167159)  16. 7 and 15 (6907)  17. limit 16 to english language (6572)  18. limit 17 to yr="2010 -Current" (4081)  19. limit 18 to (comment or editorial or letter) (118)  20. 18 not 19 (3963)  21. ((critical or evidence-based or integrative or rapid or realist or scoping or systematic or umbrella) adj (overview* or review* or synthes*)).tw,kf. (229715)  22. limit 20 to "review articles" (401)  23. 20 not 22 (3562)  24. limit 20 to (meta analysis or "systematic review") (107)  25. ((critical or evidence-based or integrative or rapid or realist or scoping or systematic or umbrella) adj (overview* or review* or synthes*)).tw,kf. (229715)  26. (metanalys* or metaanalys* or meta-analys*).tw,kf. (193217)  27. 25 or 26 (333549)  28. 20 and 27 (202)  29. 23 or 24 or 28 (3683) |  | 3683 |
| SCOPUS | ( ( TITLE-ABS-KEY ( allied AND health OR clinician* OR doctor* OR health AND profession* OR medical AND student* OR nurs* OR paremedic* OR physician* OR psycholog* OR psychiatr* OR social AND work* OR teacher* ) ) AND ( TITLE-ABS-KEY ( educat* OR train* ) ) ) AND ( ( TITLE-ABS-KEY ( ( digital AND therapeutic* OR digital AND technolog* OR ehealth OR e-health OR e-support* OR information AND technolog* OR mobile AND health OR mhealth OR m-health OR remote AND consult* OR teleconsult* OR tele-consult* OR telehealth OR tele-health* OR telemedic* OR tele-medic* OR telepsychiatr* OR tele-psychiatr* OR teletherap* OR tele-therap* ) ) ) OR ( TITLE-ABS-KEY ( ( care OR consultation* OR educat* OR healthcare OR instruction OR learning OR teaching ) W/3 ( computer* OR digital OR electronic OR online ) ) ) OR ( TITLE-ABS-KEY ( "digital econom*" ) ) OR ( TITLE-ABS-KEY ( ( education* OR information* ) W/3 technolog* ) ) ) View less |  | 121 |
| Social work Abstracts | 1. (ZU "social work education") OR TI ( ( ((allied health or clinician* or doctor* or health profession* or medical student* or nurs* or paremedic* or physician* or psycholog* or psychiatr* or social work* or teacher*) N3 (educat* or train*)) ) ) OR AB ( ( ((allied health or clinician* or doctor* or health profession* or medical student* or nurs* or paremedic* or physician* or psycholog* or psychiatr* or social work* or teacher*) N3 (educat* or train*)) ) )  2. ((((ZU "digital social work") or (ZU "digital technologies") or (ZU "digital therapeutics")) or ((ZU "telehealth") or (ZU "telemedicine"))) or ((ZU "computer-mediated communication"))) or ((ZU "online counseling") or (ZU "online social work"))  3. TI ( ( ((care or consultation* or educat* or healthcare or instruction or learning or teaching) N3 (computer* or digital or electronic or online)) ) ) OR AB ( ( ((care or consultation* or educat* or healthcare or instruction or learning or teaching) N3 (computer* or digital or electronic or online)) ) )  4. TI ( ( (digital therapeutic* or digital technolog* or ehealth or e-health or e-support* or information technolog* or mobile health or mhealth or m-health or remote consult* or teleconsult* or tele-consult* or telehealth or tele-health* or telemedic* or tele-medic* or telepsychiatr* or tele-psychiatr* or teletherap* or tele-therap*) ) ) OR AB ( ( (digital therapeutic* or digital technolog* or ehealth or e-health or e-support* or information technolog* or mobile health or mhealth or m-health or remote consult* or teleconsult* or tele-consult* or telehealth or tele-health* or telemedic* or tele-medic* or telepsychiatr* or tele-psychiatr* or teletherap* or tele-therap*) ) )  5. TI digital econom* OR AB digital econom*  6. TI ( ( ((education* or information*) N3 technolog*) ) ) OR AB ( ( ((education* or information*) N3 technolog*) ) )  7. 2 or 3 or 4 or 5 or 6  8. 1 and 7 | Scholarly (Peer Reviewed) Journals; Publication Date: 20100101-20211231 | 23 |
| Web of Science | 1. TI=((allied health or clinician* or doctor* or health profession* or medical student* or nurs* or paremedic* or physician* or psycholog* or psychiatr* or social work* or teacher*) AND (educat* or train*) )  Indexes=SCI-EXPANDED, SSCI Timespan=2010-2021  2. TI=((care or consultation* or educat* or healthcare or instruction or learning or teaching) AND (computer* or digital or electronic or online) )  Indexes=SCI-EXPANDED, SSCI Timespan=2010-2021  3. TI=((digital therapeutic* or digital technolog* or ehealth or e-health or e-support* or information technolog* or mobile health or mhealth or m-health or remote consult* or teleconsult* or tele-consult* or telehealth or tele-health* or telemedic* or tele-medic* or telepsychiatr* or tele-psychiatr* or teletherap* or tele-therap*))  4. TI=(((education* or information*) AND technolog*))  5. TI=(digital econom*)  6. 2 or 3 or 4 or 5  7. 1 and 6 |  | 942 |

**Supplementary Table 2**

*Overview of included studies*

| Author (Year)  COUNTRY | Study Aims or Objectives | Course learning objectives | | | | | | Participants | Teaching and caring technology | | | | | | | | | | | Key findings |
| --- | --- | --- | --- | --- | --- | --- | --- | --- | --- | --- | --- | --- | --- | --- | --- | --- | --- | --- | --- | --- |
|  |  | Develop digital skills | Build discipline specific remote caring skills | Create an online resource | Prepare for future remote practice | Assess students or patients online | Understand recipients experience |  | Audio/video conferencing software | Doximity Dialer applications | Online learning systems | Telehealth monitoring systems | Virtual reality technology | Telepresence robot | Virtual consultation platform | Online multimedia | Portable mobile devices | Productivity software | Simulated electronic health records |  |
| MEDICINE | | | | | | | | | | | | | | | | | | | | |
| QUANTITATIVE | | | | | | | | | | | | | | | | | | | | |
| Observational: Cross-sectional survey | | | | | | | | | | | | | | | | | | | | |
| Afonso et al. (2020)  USA | To study the effectiveness of online learning in the realm of telemedicine |  | x |  | x |  |  | First-year medical students (n=122) | x |  | x |  |  |  |  | x |  |  |  | - Students improved their telemedicine communication skills (93%) and interpretation of physical exam findings (84%) |
| Chorney & Lewis (2011)  USA | To explore the learning experiences of medical students taking an online course |  | x |  |  |  |  | Third and fourth year medical students (n=77) |  |  |  |  |  |  |  | x |  |  |  | - CORE cases made good use of students self-directed study time (84%), provided useful resources (73%), were appropriate for their level of training (86%), and expanded their knowledge and understanding of remote radiology (88%) |
| Liu et al. (2016)  AUSTRALIA | To understand the learning experience associated with an online EQClinic teaching tool to help develop online communication skills | x |  |  |  | x |  | Medical students (n=8) Simulated patients (n=3) |  |  |  |  | x |  |  |  | x |  |  | - Students felt more confident about their communication skills - Students were positive about and felt comfortable with using the technology - Students realized the importance of learning how to conduct teleconsultations to prepare for future practice - Students felt rushed during the consultation and confused about organising the structure of the consultation |
| Observational: Cross sectional | | | | | | | | | | | | | | | | | | | | |
| Sartori et al. (2019)  USA | To understand the nature of learning associated with a virtual encounter using telemedicine technology |  |  | x |  |  |  | Medical residents (n=25) |  |  | x |  |  |  |  |  |  |  |  | - Students without prior telemedicine experience struggled with telemedicine-specific assessment items - Students struggled with telemedicine-specific assessment items including remote medication reconciliation, virtual physical examination, obtaining a comprehensive description of symptoms and making appropriate plans for lifestyle or medication adjustment |
| Sartori et al. (2020)  USA | To explore the effectiveness of an online objective structured clinical evaluation tool (OSCE) to identify curriculum content gaps in telemedicine-specific skill development |  |  |  |  | x |  | Medical residents (n=78) | x |  |  |  |  |  |  |  |  |  |  | - When core communication domains were analyzed in aggregate, a mean 95% (SD 3.3%) of residents received well done within the Information Gathering domain, 91% (SD 4.9%) received well done within Relationship Development, and 78% (SD 14%) received well done within the Education/Counseling domain - Only 46% (SD 45%) of residents received well done evaluations in the Telemedicine domain with only 24% (n =19) of residents assessing technical barriers during the encounter, and 18% (n=14) attempting a virtual physical examination - Only 17% of residents (n =13) received well done evaluations for using video to augment information gathering - Self-assessments, provided by a subset of residents (n=23), demonstrated that residents felt confident with performance despite standardized patient evaluations - Most residents (91%) reported that they felt prepared for this telemedicine encounter, and only 9% (n=2) of residents reported that the encounter could have gone better |
| Experimental: Single-case (pre-post) | | | | | | | | | | | | | | | | | | | | |
| Campbell et al. (2015)  BRAZIL  MEXICO  PHILIPPINES | To gain a better understanding of the overall learning gained through online tele-education training |  | x |  |  |  |  | Ophthalmology trainees (n=81) |  |  | x |  |  |  |  |  |  |  |  | - Students reported effective use of tele-education for teaching a clinically challenging ophthalmic disease in middle-income countries - Students identified internet-based tele-education can be effective and preferred to more traditional educational models - This tele-education system significantly improved the diagnostic accuracy and reliability of ophthalmology trainees - Participants found tele-education system easy to use and learned more effectively from ROP cases with automatic, targeted error-based feedback compared to a traditional textbook format |
| Rienits et al. (2016)  AUSTRALIA | To examine the learning experience associated with an online telehealth consultation modality | x |  |  |  |  |  | Medical students (n=59) | x |  |  | x |  |  |  |  |  |  |  | - Students ratings demonstrated significant improvement both in medical student understanding of the issues and procedures, and in their confidence in conducting a telehealth consultation |
| Walker et al. (2019)  USA | To examine the level of learning associated with an online introductory telemedicine course | x |  |  |  |  |  | Second-year medical students (n=153) | x |  | x |  |  |  |  | x |  |  |  | - Students report a positive learning experience and post-test results showed higher (positive) mean scores regarding basic telemedicine knowledge and confidence - Mean telemedicine confidence scores increased from pre to post training by an average increase of 0.51 - Greatest improvement in knowledge regarding designing an office conducive for a telemedicine visits; the steps that go into a telemedicine visit; the types of telemedicine peripheral equipment; and operating telemedicine equipment |
| QUALITATIVE | | | | | | | | | | | | | | | | | | | | |
| Descriptive | | | | | | | | | | | | | | | | | | | | |
| Cannon et al. (2021)  UNITED KINGDOM | To gain a better understanding of student learning in an online focus group forum |  |  |  | x |  |  | Final year medical students (n=5) | x |  |  |  |  |  | x | x | x |  |  | - Students appreciated the opportunity to get acquainted with remote consultation platforms used in primary care |
| Thematic analysis | | | | | | | | | | | | | | | | | | | | |
| Cantone et al. (2019)  USA | To explore the effectiveness of performance feedback issued to students via an online objective structured clinical evaluation tool (TeleOSCE) | x | x |  |  |  |  | Medical students in second, third, or fourth year (n=287) | x |  |  |  |  |  |  |  |  |  |  | - Students thought the TeleOSCE was a positive, important, an challenging experience and wanted more teaching about telemedicine - Students appreciated the differences between in-person and remote visits - Students worried about how they would be judged, experienced technical limitations, and requested more time with the technology - Some students viewed telemedicine as a triage measure |
| Darnton et al. (2020)  UNITED KINGDOM | To explore the benefits associated with online consultations and their role in advancing medical practice |  |  |  | x |  |  | Medical students in second year (n=13)  General practitioners (n=10) | x |  |  |  |  |  | x |  | x |  |  | - Students gained a unique perspective and appreciation of how to conduct remote medical care for their future practice - Intentional approach towards addressing technology challenges |
| MIXED METHODS | | | | | | | | | | | | | | | | | | | | |
| Abraham et al. (2020)  USA | To understand the ways in which telehealth may augment physician-related care | x | x |  | x |  |  | Third-year medical students (n=20) | x | x |  |  |  |  |  | x |  |  |  | - 90% of students valued the telemedicine experience and appreciated the benefits for their future practice - 80% felt that telehealth would play an important role in their future careers - All students identified the value of telehealth service for their patients - 93% felt that doctors can provide quality care using telehealth technology |
| Bulik & Shokar (2010)  USA | To examine the effectiveness of learning within an online telehealth course | x |  |  |  |  |  | Fourth-year medical students (n=7) | x |  |  |  |  |  |  |  |  |  |  | - Students valued their telemedicine experience, regarded it as an effective learning experience, and would recommend it to their peers |
| Martinez et al. (2020)  USA | To understand the effectiveness of learning within an online telemedicine clinical skills course | x |  |  |  | x |  | Medical students (n=47) |  |  |  | x |  |  |  |  |  |  |  | - Students felt telemedicine was relevant to their medical education and enforced the importance of the physical exam - A large subset of students felt that the sessions helped improve their ability to acquire pertinent medical information over a video platform - Telemedicine highlighted the difficulties in engaging with patients over a video platform |
| Newcomb et al. (2021)  USA | To explore the learning experiences of students as they engage with telehealth technology and simulated patient scenarios | x |  |  |  |  |  | N=11  Fourth-year medical students (n=5)  Observers (n=4)  Faculty (n=2) | x |  |  |  |  |  |  |  |  |  |  | - Students reported increased comfort with working with telehealth technology - Students appreciated the ability to gain more knowledge about virtual communication and how to engage with the simulated patient - Students appreciated the opportunity for direct observation of skills and immediate faculty feedback |
| EDUCATION | | | | | | | | | | | | | | | | | | | | |
| QUALITATIVE | | | | | | | | | | | | | | | | | | | | |
| Inductive | | | | | | | | | | | | | | | | | | | | |
| Jang & Chen (2010)  TAIWAN | To investigate the impact of transformative teaching model to enhance the teaching knowledge of science-based student teachers as they use technology |  | x |  |  |  |  | Pre-service teachers in second year of teacher education program (n=12) |  |  | x |  |  |  |  |  |  |  |  | - Pre-service teachers learned how to integrate technologies with teaching through transformative model and web-based learning environment - Pre-service teachers believed the transformative learning model would shape their future teaching practice |
| Interpretative | | | | | | | | | | | | | | | | | | | | |
| Forbes & Khoo (2015)  NEW ZEALAND | To explore potentially innovative and creative teaching and learning experiences during online podcast development |  |  |  | x |  | x | Lecturer (n=1) Tutors (n=2) Students (n=60) |  |  | x |  |  |  |  | x |  |  |  | - Participants appreciated the innovative and creative pedagogical aspects of this project - Findings indicate the importance of a role-modeled co-learning process (as teachers created their own podcasts) as well as learning new skills and technologies |
| Grounded Theory | | | | | | | | | | | | | | | | | | | | |
| O'Connor & Worman (2019)  USA | To explore the learning experience with virtual reality as a teaching modality | x |  |  |  |  |  | Teacher-education students (n=12) |  |  |  |  | x |  |  |  |  |  |  | - Majority of students reported overall positive experiences. Struggles with language and terminology improved over time with computer-mediated experiences - Technical difficulties and limited ability of students within virtual reality spaces were reported as the major challenges |
| Satar & Akcan (2014)  TURKEY | To understand the level of acceptance associated with online teaching and learning tools |  |  |  |  | x |  | Pre-service English language teachers (n=42) |  |  | x |  |  |  |  | x |  |  |  | - The students were willing to learn about the use of new technologies in teaching - Online tutoring skills training enhanced pre-service teachers’ awareness towards the active use of the online platform - Recommendations for designing of blended courses based on the workload of tutors are suggested - Difficulties related to higher number of online posts, design and topics of the tasks, student preferences for passive participation have been mentioned - Further research on the interrelationship between task design and participation in blended learning settings is suggested |
| MIXED METHODS | | | | | | | | | | | | | | | | | | | | |
| Chittleborough (2014)  AUSTRALIA | To explore the impact that online learning has on pre-service teacher preparedness to teach in an online format | x |  |  | x |  |  | Third year of Bachelor of Science /Bachelor of Teaching program  (n =18)  Master of Teaching students (n=10) |  |  | x |  |  |  |  | x | x | x |  | - Students appreciated online learning experience and the opportunity to learn transferable skills - Some pre-service teachers mentioned the online learning process to be tedious and could not appreciate the opportunity of using it in schools in near future and by the time it is available and reliable, they thought they would need to relearn how to use it |
| He (2014)  USA | To explore the impact of online teacher education course on pre-service teachers’ perceptions of challenges and advantages |  |  |  |  |  | x | Teacher candidates (n=24)  Graduate students (n=22)  International exchange students (n=2) |  |  | x |  |  |  |  |  |  |  |  | - Students experienced an increase in their confidence levels and self -efficacy in teaching and learning - Promoting assessment and feedback efforts at the institutional level could further extend our understanding of online instruction and offer learners more opportunities to contribute to the improvement of online course offerings |
| Lan et al. (2012)  TAIWAN | To explore the awareness of gaps between theory and practice using a synchronous online teaching model that facilitates teacher skill development in online teaching forums |  |  | x |  |  |  | Pre-service teachers (n=46) |  |  | x |  |  |  |  |  |  |  |  | - Students enhanced their online teaching skills and experienced an increase in cognition and action skill development - Improved awareness of the benefits of self-reflection were also reported |
| Luo et al. (2017)  USA | To explore the effect of a pre-service teacher-developed online course that includes authentic learning content |  |  | x | x |  |  | Student teachers (n=48, 28 males, 20 females) |  |  | x |  |  |  |  | x |  |  |  | - Technologies used helped to enhance students’ technical competencies, while improving upon methods to create educational content in the future - Pre-service teachers were likely to teach and/or develop online learning opportunities in the future - More emphasis is needed to expose pre-service teachers to learning management systems and digital online collaboration tools to prepare them as online educators |
| Russo & Siko (2018)  USA | To explore the probability of student teachers using technology-infused teaching modalities in their lesson plans after having experienced learning within an online course | x |  |  |  |  |  | Pre-service teachers (n=13) |  |  | x |  |  |  |  |  |  | x |  | - Student resource use supported the idea of learning through technology being superior to simply providing students a one-off exercise and referencing online course materials - Better mechanisms are needed o ensure pre-service teachers' use technology across all subject areas during their placements consistent with current technology standards - Field coordinators and cooperating teachers highly skilled in technology integration could act as conduits for further confidence building and increasing effective technology integration abilities - More longitudinal studies are needed to examine persistence of pre-service teachers' pedagogical practices of incorporating technology in their future teaching at various intervals selected to span changes in specific technology tools available |
| Sepulveda-Escobar & Morrison (2020)  CHILE | To examine the advantages and disadvantages associated with online learning within a teaching placement course | x |  |  |  |  |  | Student teachers (n=27) | x |  |  |  |  |  |  | x |  |  |  | - Lack of interaction which might affect student professional, social, and personal development - Lack of communication with the supportive agents affected the feedback students received, and the possible benefits of it on their future work - Need to actively enquire into the student teachers’ experiences during online placements in order to support teacher development - Need to generate suitable strategies for online teaching placements as an opportunity to continue learning - Identifying and explore the distinctive aspects of the virtual experience during the pandemic |
| Tømte et al. (2015)  NORWAY  SWEDEN | To explore how online teachers may facilitate digital competencies in student teachers and discover the potential innovation with online teaching and learning | x |  |  | x |  |  | Student survey (n =155)  Student interviews  ( n =41)  Teacher interviews  (n =16)  School staff interviews  (n = 15) | x |  | x |  |  |  |  |  |  |  |  | - Online education programmes stimulate student teachers to develop digital competence skills - Levels of teacher confidence associated with integrating online approaches into the classroom represented a barrier for integration |
| Woodcock et al. (2015)  AUSTRALIA | To investigate the effects that an online learning platform (eLearning) has on the feelings of readiness to teach in pre-service teachers |  |  |  |  | x |  | Pre-service teachers (n=53) 25% males 75% females |  |  | x |  |  |  |  | x |  | x |  | - E-learning increased teacher self-efficacy and competence related to learning in a psychologically safe environment besides increasing their sense of accountability for learning - Participants stated that they were able to learn from home, participate anonymously, and feel more confident to ask questions for understanding, and, thus, were more comfortable sharing their ideas in a judgment-free environment - Participants reported difficulty to concentrate online and mentioned that online errors reduced their confidence - Need to develop a framework for understanding the conditions that must be met sequentially in order to enhance e-learning competence including competence, e-learning efficacy, psychologically safe environments, and ease of use |
| NURSING | | | | | | | | | | | | | | | | | | | | |
| QUALITATIVE | | | | | | | | | | | | | | | | | | | | |
| Thematic analysis | | | | | | | | | | | | | | | | | | | | |
| Posey et al. (2020)  USA | To understand the learning experience associated with telehealth simulated patient encounters |  |  |  |  | x |  | Nurse practitioner students (n=18) | x |  |  |  |  |  |  |  |  |  |  | - Although students expressed a lack of familiarity and comfort with the telehealth and standardized patient encounters, they valued the standardized patient simulations in preparing for clinical practice - Students valued practicing clinical and telehealth skills outside of a high-stakes testing environment - The virtual encounter was valuable in developing telehealth skills in preparation for future practice - Students desired more formative standardized patient simulations within the NP curriculum |
| MIXED METHODS | | | | | | | | | | | | | | | | | | | | |
| Lister et al. (2018)  USA | To explore an innovative online learning experience with the use of a telepresence robot, video-conferencing technology and simulated patients |  | x |  |  |  |  | Third-year nursing students (n=73) | x |  |  |  |  | x |  |  |  |  |  | - Overall improvement in students' confidence in their ability to communicate via video technology, as well as improved opinion of video technology's value in delivery of healthcare - 45% of students said the telepresence robot facilitated long-distance care, 15% said the simulation introduced them to new technologies and 24% said it promoted communication - 60% of students responded that their inability to touch the patient changed their nursing care, 23.6% said it changed the nursing care somewhat, and 16.4% denied it affected their care - Students identified the simulation as realistic and the teaching methods as helpful and effective - 41.5% of students found their assessment capabilities limited by the use of telepresence, 35.8% said the interaction felt impersonal, and 17% found technical problems interfered with the interaction - 20.5% of students would have liked to improve their communication techniques, 12.8% would have like to perform more patient education, and 10.2% noted they would prefer enhancements made to the technology used |
| Love & Carrington (2020)  USA | To explore the learning experiences of students as they engage with telehealth technology and simulated patient scenarios | x |  |  |  |  |  | Doctor of nursing students (n=83)  Simulated patients (n=12) |  |  | x |  |  | x |  | x |  |  |  | - 16% of students felt prepared to interview the patient and 17.5% felt prepared to lead an interprofessional collaborative meeting - Human factors or user-technology issues, inability to perform a “hands-on” examination and reliance on the patient’s senses for health examinations were reported barriers to technology integration - Simulated patients expressed similar perceptions of missing human component of being in the office |
| Pullen & Silvers (2018)  USA | To investigate the effectiveness of online high impact teaching strategies for skill development with communication and safety practices | x |  |  | x |  |  |  | x |  | x |  |  |  |  |  |  |  |  | - High course success rates with students reporting improved communication skills - Employers indicated graduates exceeded expectations in the use of high impact teaching strategies - Nurse leaders in healthcare organizations and nurse educators in higher educational settings need to collaborate and identify high impact teaching competencies and learning experiences to prepare graduates for practice |
| Quinlin et al. (2020)  USA | To explore the effectiveness of an objective structured clinical evaluation tool (OSCE) designed to evaluate student learning with online telehealth learning opportunities |  | X |  |  | x |  | Family nurse practitioner students (n=103) | x |  |  |  |  |  |  |  |  |  |  | - All students were successful in introducing and concluding the visit and were able to demonstrate proficient use of the technology - Students who completed the telehealth examination had increased confidence in their decision-making and readiness to provide care by telehealth - Students valued the telehealth experience, the feedback from faculty, and the dynamic interaction that occurred during the group debriefing - Cost in terms of time, energy, and efforts were reported barriers in technology integration |
| Rutledge et al. (2020)  USA | To understand more about the learning experience associated with taking an online telehealth course | x |  |  | x |  |  | Students from seven different allied health professions (n=67) |  |  | x |  |  |  |  | x |  |  |  | - Program improved students confidence in planning and provision of telehealth. Score of the Telehealth Etiquette Knowledge scale increased significantly after the program - MD students had significantly lower preprogram scores on the Telehealth Etiquette Knowledge scale than the non-MD students - Students appreciated the telehealth rotation being provided at this crucial time, accepted the role of telehealth in the future, increased their understanding and awareness of the impact of telehealth and the different roles of professions and how they applied to telehealth - The realism of the program was relevant and underscored the importance of telehealth - Faculty learned how a program that was previously hybrid in delivery could be successfully implemented and offered completely online while being as effective or even more so than its previous iteration |
| KINESIOLOGY | | | | | | | | | | | | | | | | | | | | |
| QUANTITATIVE | | | | | | | | | | | | | | | | | | | | |
| Experimental: Single-case (pre-post) | | | | | | | | | | | | | | | | | | | | |
| Winkelmann & Eberman (2020)  USA | To explore the level of learning associated with engaging with standardized patients through a simulated telehealth visit |  |  |  |  | x |  | Second-year master’s Athletic Training students (n=55) | x |  | x |  |  | x |  | x | x |  |  | - Overall, the mean sum confidence score across 18 items reported by the participants improved from 68.41 6 8.13 at preintervention intervention to 69.35 6 9.44 (of 90) measured on the postintervention survey - Significant decrease in confidence for using appropriate professional language when interacting with patients (p=.026) - Significant improvement in confidence at the .01 a level for using telemedicine technology (p<=.001) - The use of a standardized patient (using telepresence robot instrumentation) significantly improves the athletic training students' confidence in using telemedicine technology |
| SOCIAL WORK | | | | | | | | | | | | | | | | | | | | |
| QUANTITATIVE | | | | | | | | | | | | | | | | | | | | |
| Observational: Cross-sectional survey | | | | | | | | | | | | | | | | | | | | |
| Goldingay & Body (2017)  AUSTRALIA | To explore how online learning prepares students for technological /digital advancements in their future practice | x |  |  | x |  |  | Bachelor of social work students (n=26)  Master of Social Work students (n=75) |  |  | x |  |  |  |  |  | x |  |  | - Students learned how to interact online in a respectful manner with integrity - Clear guidelines on how to interact in a respectful, appropriate, and considered manner helped students develop skills for competent, safe, and sensitive online engagement - The inbuilt community of practice provide the framework for future online mediated interactions they in future practice |
| INTERDISCIPLINARY | | | | | | | | | | | | | | | | | | | | |
| QUALITATIVE | | | | | | | | | | | | | | | | | | | | |
| Content analysis | | | | | | | | | | | | | | | | | | | | |
| Estes et al. (2016)  USA | To explore the level of learning associated with engaging with simulated patients through a simulated telehealth visit | x | x |  |  |  |  | Students from a nursing program and pharmacology program (n=15) | x |  |  | x |  |  |  |  |  |  | x | - Students appreciated the combined effect of using telehealth technology and working through an interprofessional simulated telehealth scenario - Students recognized the ability to increase connectivity and communication between the patient and the provider in an interprofessional manner despite distance - Simulated telehealth technology can be implemented into curriculum and across disciplines to facilitate the learning as it applies to the clinical context |
| MIXED METHODS | | | | | | | | | | | | | | | | | | | | |
| Bautista et al. (2020)  USA | To understand ways that collaborative care can be enhanced through online teaching and learning strategies |  | x |  | x |  |  | Medical students (n=2) Pharmacy students (n=3) | x | x |  |  |  |  |  |  |  |  |  | - Students experienced increased confidence levels associated with collaborative care approaches gained through the online learning experience - Students identified the advantages associated with using telemedicine in a clinical rotation to serve as a model for future interprofessional education opportunities |
| Nearing et al. (2020)  USA | To understand the need for telehealth training in education program development |  |  |  |  | x |  | N=89  Associated health trainees (n=70)  Medicine fellows (n=15)  Advanced research fellows (n=4) |  |  |  |  |  |  |  |  |  |  |  | - Students with prior telehealth experience reported a higher level of comfort compared to those with no experience - Most participants expressed that the video-to-home visits were an extremely part of the COVID-19 response and 35% indicated that they were “extremely likely” to incorporate these visits into their future practice - In-person telehealth training with a supervisor/preceptor was indicated as a valuable future opportunity - Desire for training and resources that focused on nuts-and bolts issues, such as equipment/software, establishing the necessary infrastructure to do video visits from home, and how to address issues such as scheduling and billing |

**Supplementary Table 3**

*Quality appraisal scores for quantitative studies*

| **Authors (year)** | **Quantitative Study design** | **Selection Bias** | **Design** | **Confounders** | **Blinding** | **Data Collection** | **Withdraw/drop-outs** |
| --- | --- | --- | --- | --- | --- | --- | --- |
| Afonso et al. (2020) | Observational: Cross-sectional survey | W | W | W | W | W | W |
| Abraham et al. (2020)* | Experimental: Single-case (pre-post) | W | W | W | W | W | W |
| Bautista et al. (2020)* | Observational: Cross-sectional survey | W | W | W | W | W | W |
| Bulik & Shokar (2010)* | Observational: Cross-sectional survey | W | W | W | W | W | W |
| Campbell et al. (2015) | Experimental: Single-case (pre-post) | W | W | W | W | S | W |
| Chittleborough (2014)* | Observational: Cross-sectional survey | W | W | W | W | W | W |
| Chorney & Lewis (2011) | Observational: Cross-sectional survey | W | W | W | W | W | W |
| Goldingay & Boddy (2017) | Observational: Cross-sectional | W | W | W | W | W | W |
| He (2014)* | Experimental: Single-case (pre-post) | W | M | W | W | M | W |
| Lan et al. (2012)* | Observational: Cross-sectional survey | W | W | W | W | W | W |
| Lister et al. (2018) | Experimental: Single-case (pre-post) | M | W | W | W | S | W |
| Liu et al., 2016 | Observational: Cross-sectional survey | M | W | W | W | W | M |
| Love & Carrington (2020)* | Observational: Cross-sectional survey | M | W | W | W | W | S |
| Luo et al. (2017)* | Experimental: Single-case (pre-post) | W | W | W | W | W | W |
| Martinez et al. (2020)* | Observational: Cross-sectional survey | M | W | W | W | W | W |
| Nearing et al. (2020)* | Observational: Cross-sectional survey | M | W | W | W | W | W |
| Newcomb et al. (2021)* | Experimental: Single-case (pre-post) | S | W | W | W | W | W |
| Quinlin et al. (2020)* | Observational: Cross-sectional | W | W | W | W | W | W |
| Pullen & Silvers (2018) | Observational: Cross-sectional survey | W | W | W | W | W | W |
| Rienits et al. (2016) | Experimental: Single-case (pre-post) | W | W | W | W | W | W |
| Russo & Siko (2018)* | Observational: Cross-sectional survey | W | W | W | W | W | W |
| Rutledge et al. (2020)* | Experimental: Single-case (pre-post) | M | M | M | W | M | W |
| Sartori et al. (2020) | Observational: Cross-sectional | W | W | W | W | W | W |
| Sartori et al. (2019) | Observational: Cross-sectional | W | W | W | W | W | W |
| Sepulveda-Escobar (2020)* | Observational: Cross-sectional survey | W | M | W | W | M | W |
| Tømte et al. (2015)* | Observational: Cross sectional survey | W | W | W | W | S | W |
| Walker et al. (2019) | Experimental: Single-case (pre-post) | W | M | W | W | M | S |
| Winkelmann & Eberman (2020) | Experimental: Single-case (pre-post) | M | M | S | W | S | S |
| Woodcock et al. (2015)* | Experimental: RCT | S | W | W | W | W | W |

*Note:* *indicates mixed methods study; S, strong; M, moderate; W, weak; N/A, not applicable

**Supplementary Table 4**

*Quality appraisal scores for qualitative studies*

| **Authors (Year)** | **Qualitative**  **Study Design** | **Philosophy congruent** | **Objective Congruent** | **Data collection congruent** | **Data analysis congruent** | **Interpretation of results** | **Theory or cultural location** | **Researcher reflexivity** | **Participant representation** | **Ethical consideration** | **Conclusion** |
| --- | --- | --- | --- | --- | --- | --- | --- | --- | --- | --- | --- |
| Abraham et al. (2020)* | Thematic analysis | U | Y | Y | U | N | N | N | N | N | Y |
| Bautista et al. (2020)* | Descriptive | U | Y | Y | N | N | N | N | Y | N | U |
| Bulik & Shokar (2010)* | Descriptive | U | Y | Y | N | N | N | N | Y | Y | U |
| Cannon et al. (2021) | Descriptive | U | Y | Y | U | Y | N | N | Y | N | U |
| Cantone et al. (2019) | Thematic analysis | U | Y | Y | Y | Y | N | N | Y | Y | U |
| Chittleborough (2014)* | Descriptive | Y | Y | Y | Y | Y | Y | Y | Y | N | Y |
| Darnton et al. (2020) | Thematic analysis | U | Y | Y | Y | Y | N | N | Y | Y | Y |
| Estes et al. (2016) | Content analysis | U | Y | Y | U | U | N | N | Y | N | N |
| Forbes & Khoo (2015) | Interpretive | U | Y | Y | Y | Y | N | N | Y | Y | Y |
| He (2014)* | Descriptive | N | Y | Y | U | U | N | N | Y | N | U |
| Jang & Chen (2010) | Inductive | N | Y | Y | Y | Y | N | N | Y | N | Y |
| Lan et al. (2012)* | Grounded theory | U | U | U | U | U | N | N | Y | N | U |
| Lister et al. (2018)* | Content analysis | U | U | U | U | U | N | N | Y | Y | Y |
| Love & Carrington (2020)* | Descriptive | U | U | U | U | U | N | N | N | N | Y |
| Luo et al. (2017)* | Descriptive | Y | Y | Y | Y | Y | N | N | Y | N | Y |
| Martinez et al. (2020)* | Descriptive | U | U | U | U | U | N | N | Y | Y | Y |
| Nearing at al. (2020)* | Thematic analysis | U | U | U | U | U | N | N | Y | N | Y |
| Newcomb et al. (2021)* | Descriptive | U | U | U | U | U | N | N | Y | N | Y |
| O'Connor & Worman (2019) | Grounded theory | Y | Y | Y | Y | Y | N | N | Y | Y | Y |
| Posey et al. (2020) | Thematic analysis | Y | Y | Y | Y | Y | Y | Y | Y | Y | Y |
| Pullen & Silvers (2018) | Descriptive | U | Y | Y | U | Y | N | N | N | N | U |
| Quinlin, et al. (2020)* | Descriptive | U | Y | Y | Y | Y | N | N | Y | N | Y |
| Russo & Siko (2018) | Descriptive | N | Y | Y | Y | N | N | N | N | Y | Y |
| Rutledge, et al. (2020)* | Descriptive | N | Y | Y | Y | Y | N | N | N | Y | Y |
| Satar & Akcan (2014) | Grounded theory | Y | Y | Y | Y | Y | Y | Y | N | Y | Y |
| Sepulveda (2020)* | Thematic analysis | Y | Y | Y | Y | Y | Y | N | N | Y | Y |
| Tømte et al. (2015)* | Descriptive | U | Y | Y | Y | U | Y | N | N | N | Y |
| Woodcock et al. (2015)* | Content analysis | N | Y | Y | Y | Y | N | N | Y | Y | Y |

*Note:* *indicates mixed methods study, Y, yes; N, no; U, unclear; N/A, not applicable.
